# The medium-term perceived impact of work from home on life and work domains of knowledge workers during COVID-19 pandemic: A survey at the National Research Council of Italy

**DOI:** 10.3389/fpubh.2023.1151009

**Published:** 2023-03-10

**Authors:** Antonella Bodini, Carlo Giacomo Leo, Antonella Rissotto, Pierpaolo Mincarone, Stanislao Fusco, Sergio Garbarino, Roberto Guarino, Saverio Sabina, Egeria Scoditti, Maria Rosaria Tumolo, Giuseppe Ponzini

**Affiliations:** ^1^Institute for Applied Mathematics and Information Technologies “E. Magenes”, National Research Council, Milano, Italy; ^2^Institute of Clinical Physiology, National Research Council, Lecce, Italy; ^3^Training and Welfare Unit, National Research Council, Rome, Italy; ^4^Institute for Research on Population and Social Policies, National Research Council, Brindisi, Italy; ^5^Department of Neurosciences, Rehabilitation, Ophthalmology, Genetics and Maternal/Child Sciences, University of Genoa, Genoa, Italy; ^6^Department of Biological and Environmental Sciences and Technology, University of Salento, Lecce, Italy

**Keywords:** knowledge workers, life domain, work domain, perceived impact, forced work from home, smart work

## Abstract

**Objective:**

The study aimed to investigate perceptions and determinants of the overall impact on life and work domains among a community of knowledge workers after 18 months of forced work from home due to the pandemic.

**Methods:**

A cross-sectional study with a retrospective assessment was conducted early in 2022 at the National Research Council of Italy. Five single-item questions explored the perceived impact on life domain while a 7-item scale the impact on the work domain. Bivariate analyses and multivariate regressions were used to evaluate the associations between impacts and some key factors defined by 29 *ad hoc* closed questions.

**Results:**

More than 95% of the 748 respondents reported a perceived change in at least one item of the life domain. For each of these items, although a large group of subjects has reported that working from home had no impact (from 27 to 55%), in the rest of the sample the positive evaluation (from 30 to 60%) clearly prevailed over the negative one. Overall, most of the subjects (64%) rated the impact on the work experience positively. Relationship with colleagues and participation in the work context were the items where the greatest number of negative rates was concentrated (27 and 25%, respectively). On the other hand, positive perceptions prevailed over both negative perceptions and lack of impact perceptions on the subjects of organizational flexibility and quality of work. The frequency of work-room sharing, home-work commute time and changes in sedentary lifestyle, have been identified as common explanatory factors of perceived impacts on both domains.

**Conclusion:**

Overall, respondents reported positive rather than negative perceived impacts of forced work from home in both their lives and work. The obtained results suggest that policies to promote the physical and mental health of employees, strengthen inclusion and maintain a sense of community are necessary to improve workers' health and prevent the effects of perceived isolation on research activities.

## 1. Introduction

In Italy, the first official COVID-19 case locally acquired was detected on 20 February 2020 in Lombardy and the rapid growth of infections prompted the Italian government to impose a first localized lockdown as early as February 23, (1). From 23 February to 11 March, more restrictive measures were introduced throughout the national territory, including the suspension of non-essential production activities and home working as an exception to legal obligations, in the public and private sectors (Decrees Law of Italian Presidency of the Council of Ministers of 23 February and 11 March 2020). The succession of pandemic waves and the related restrictions have led to the extension of smart working by way of derogation until 31 October 2021. The generic term “work from home” (WFH) is therefore more suitable than the more common term “smart-work” to describe the situation. In fact, key features such as spatial and temporal flexibility and the opportunity to achieve work-life balance from organizational flexibility have been hampered by public health measures.

In Italy, the smart working was in its first steps shortly before the pandemic, both in terms of regulating laws and application. In 2019, 81.7% of Italian employees worked mainly in premises or offices made available by the employer. Out of an estimated 7 million workers with a profession that can be exercised remotely under ordinary conditions, only 0.8% of employees had a teleworking contract (strictly regulated since 1998) or a smart working contract, (2). Furthermore, only 3.6% of Italian public institutions had implemented the Directive n. 3 of 2017 introducing smart work, (3). This was in line with the European trend, given that 9% of employed people in the EU-27 worked from home at least once in 2019 and only 5.4% regularly, with high cross-country differences, (4). Hence, the forced transition to WFH was a new experience for virtually all workers, who often lacked the basic tools and training necessary to consider working from home as authentically smart (5).

Before the pandemic, the benefits and disadvantages of flexible forms of work both for employers and employees have been assessed considering primarily work-related outcomes rather than workers' health and wellbeing (6, 7). The pandemic and the consequent worldwide use of the WFH as a containment measure, have introduced a new and disruptive element in this investigation process. The focus has shifted more often to the assessment of the impact on workers' health and wellbeing, mainly in association with the first periods of lockdown (e.g., (8–12)). This approach, often based on preliminary or partial analyses of large data collections, contributed to the important purpose of providing recommendations and guidelines to both workers and employers to better and timely address the emergency situation (7, 13). A study with similar aims was also conducted at the Italian research institutions in the spring of 2020, (14). However, at that time the fears and expectations regarding the pandemic could have influenced the perceived impact of forced WFH on life and work domains (14, 15). Enough time has passed since the initial emergence phase and these confounding effects should have reduced their influence, so as to make possible a reflection on forced WFH that could also be valid for agile working after the pandemic.

With this in mind, we designed a survey among employees of the largest public research body in Italy, the National Research Council (Consiglio Nazionale delle Ricerche, CNR). Before the pandemic, the CNR had not yet introduced forms of agile work on a large scale. The aim of this study was 2-fold: (1) to explore the medium-term perception of the change in quality of life and work experience that occurred during the entire period of working from home from February 2020 to October 2021 compared to the previous situation and (2) to identify factors associated with this perception. The analyses conducted in this study can serve as a useful tool to identify critical areas in remote work environments. The study will provide managers of CNR and similar public institutions with valuable experiential knowledge, enabling them to formulate new practices or improve existing to support the health and wellbeing of smart-workers in the post-pandemic work organization.

## 2. Material and methods

### 2.1. Study population and work organization

CNR has over 8.500 employees (47% female) who belong to 88 Institutes distributed throughout Italy, including the islands. With the exclusion of managers, employees are classified into 4 professional profiles: researcher (51%), technologist (9%), administrative (10%) and technician (27%, source CNR).

The CNR, together with other public research bodies, is part of the Italian public administration. The organization of the work of researchers and technologists is then largely different from that of university professors, while the differences are minor for technical and administrative staff. Each employee is assigned to a workplace (an Institute) and face-to-face work was the ordinary way of working before the pandemic. The working hours of the technical and administrative staff are spread over 5 days and 36 h a week, with limited flexibility. Researchers and technologists can independently manage their working time, but still referring to 5 days and 36-h week. Moreover, these latter are not subject to any hierarchical supervision of their research activity. Researchers are not required to carry out teaching activities. Before the pandemic, only a small percentage of workers (around 5%) was recurring to teleworking (the number of possible positions was 2% until 2018, raised to 10% in 2019) or part-time (3.3%). At the start of the WFH, therefore, the CNR had to face on a large scale the need to provide IT support such as computers, mobile devices or other equipment, software for secure remote access to institutional resources (databases, bibliographic resources), software for meetings and all the related training.

After the end of the emergency (end of 2021), regulated smart working was introduced for all employees, up to a maximum of 10 days a month.

### 2.2. Survey

We designed a cross-sectional study with a retrospective assessment among all CNR permanent workers hired before 1 June 2019. An online individual questionnaire was administered, through a dedicated server managed by a private company (eResult s.r.l.) which acted as an external processor pursuant to the Regulation (EU) 2016/679. Data was collected using the LimeSurvey open source tool (Community Edition version 3.26.1). The invitation was sent by e-mail to the mailing list including all the employees, with the authorization of the CNR General Manager. The survey started on 12 January 2022 and was closed on 9 March 2022. Up to 3 follow-up emails reminded employees to take the survey.

The survey was developed by researchers in the fields of public health, health and wellbeing, work-related stress and statistical methods. Subjects were asked to directly report their perception of the impact of WFH on their life domains through 5 questions “According to your perception, how the experience of working from home has affected your (Q1) lifestyle, eating habits and health status; (Q2) quantity/quality and disturbances of sleep, and daytime sleepiness; (Q3) psychological status; (Q4) quality of interpersonal relationships within the family; (Q5) quality of interpersonal relationships within the network of friends.” A 5-point Likert-type scale from 1 (very negatively) to 5 (very positively) was used.

As far as changes in the work domain are concerned, subjects were asked to report their perception of the impact of WFH on their work experience by using a 7-item scale: “According to your perception, how the experience of working from home has affected your (i) ability to take initiatives and propose solutions in the workplace; (ii) participation in the working context; (iii) relationship with colleagues; (iv) relationship with superiors; (v) quality of work; (vi) organization of personal environment and workspace; (vii) management of the working time. The same 5-point Likert-type scale reported above was adopted.

A total of further 29 closed questions investigated socio-demographic data, individual factors [related to hobbies and pastimes, time spent on walking, on vigorous and moderate physical activity, (16)], family factors (e.g., size of the house, number of family members sharing the same accommodation, the number and age of children in the household and the presence inside and outside the home of people in need of assistance), and individual organizational factors related to the working space available in the home. Moreover, the survey included a few clinical questionnaires validated for the Italian population (MeDAS (17), PSQI (18), ESS (19), MEQr (20) and PHQ (21, 22). All of these questionnaires, except the MEQr, were asked to be filled in referring both before and during the WFH period.

The questionnaire was organized in four sections and took about 40 min to be completed. With the aim of encouraging a large participation, only section 1 investigating socio-demographics, individual and family factors together with the work domain was fully mandatory.

A first version of the questionnaire was pretested to verify the clarity of the terminology, the absence of ambiguity, the completeness of the alternative answers, the absence of inadequate or privacy-damaging questions, the possible presence of questions deemed unnecessary as well as the ease of use of the administration tool. Twenty subjects from the target group were involved on a voluntary basis and were asked to provide a detailed opinion on each *ad-hoc* question, the questionnaire as a whole and on the encountered technical difficulties. The questionnaire was then refined according to the results of the pilot phase.

This analysis focuses on one of the purposes of the general study, and other aspects will be discussed in dedicated articles.

### 2.3. Ethical issues

The study was conducted for research purposes only, in accordance with the 1964 Helsinki declaration and ethical approval was provided by the CNR Research Ethics and Integrity Committee, on October 28, 2021 (Ethical Clearance 0078918/2021). The invitation email was sent directly by the principal investigators of the study. The purpose of the research as well as all the precautions taken to ensure confidentiality and data protection have been clearly explained in the email. Participation was voluntary, without compensation. Only a few of the authors had access to the gathered data, including participation, that were not communicated to the CNR Administration. Informed consent was a prerequisite for participation. A conservation period of 3 years has been fixed for data verification during publication, after that data and their digital copies will be deleted. On the meanwhile, the filled questionnaires are kept in a locked file.

### 2.4. Statistical analysis

Descriptive statistics included crude and relative frequency data and location-scale summaries. Frequencies were aggregated in case of very low values. The frequency of the option “I don't know” for each item was computed (from 0.8 to 5.1%) and imputation based on the most frequent response was applied.

Bivariate analysis was based on both the chi-square test and the non-parametric Wilcoxon test and Kruskal-Wallis test (with the Bonferroni correction for multiple testing).

#### 2.4.1. Life domain analysis

For inference purposes, the very negative (very positive) and negative (positive) responses to (Q1)–(Q3) were merged as the extreme frequencies were very low. A first cross-check on the reliability of the (merged) responses to (Q1)–(Q3) based on the total score variations of the clinical questionnaires (MeDAS for the lifestyle, PSQI and ESS for sleep quality, PHQ for depressive status) was made by the Kruskal-Wallis test.

The total score variations of MeDAS, PSQI, ESS and PHQ were then classified in terms of worsening (decrease in MeDAS score, increase in PSQI, ESS, and PHQ scores), no change, and improvement (increase in MeDAS score, decrease in PSQI score, ESS and PHQ scores) and a further cross-check was made by the chi-square test on the 3 × 3 contingency tables of self-reported rates vs. measured variations. Consistency was established if the standardized residuals on the main diagonals of the significant contingency tables were all positive and significant with respect to the quantiles of a standard normal distribution.

Finally, a univariable multinomial regression analysis was carried out to select the variables to be included in a full multivariable multinominal logistic regression for the outcomes from Q1 to Q5. The no perceived impact group was considered as the reference group. Any variable whose univariable test had *p* < 0.20 was included in the multivariable model. Stepwise model selection by AIC was used to identify a final, parsimonious model and determine effect measures in the form of adjusted odds ratios (ORs) and 95% CIs of perceived positive/negative impact of WFH vs. no perceived impact on the life domain. The adjusted generalized variance-inflation factor [aGIF = GVIF^∧^(1/(2×df)] with the conservative vif-threshold of 5 was computed to deal with multicollinearity and further refine the model up to the definition of the main effect model (23).

#### 2.4.2. Work domain analysis

Cronbach's alpha and Guttman's λ_4_ and λ_6_ were computed on the imputed data. The average inter-item correlation was 0.54, very close to the median inter-item correlation of 0.53. The reliability coefficients were Cronbach's Alpha = 0.89 (95% CI: 0.87–0.90), λ_4_ = 0.91 and λ_6_ = 0.89. Leave-one-out item reliability ranged from 0.86 to 0.88 for both Cronbach's Alpha and λ_6_. Homogeneity of the items was also confirmed by the ICLUST algorithm, indicating one only cluster of the seven items. The existence of one only latent trait underlying the data was investigated by a confirmatory factorial analysis with one factor. Although the calculated indices did not provide a univocal indication of one-dimensionality (χ^2^ = 292.351 with 14 df, root mean square residual of 0.082, 90% CI of the root mean square error of approximation from 0.147 to 0.180), high values of the Comparative Fit Index (0.985) and of the Tucker-Lewis Index (0.977) suggested a good model fit. The 7 scores were then averaged to form a composite measure of the impact of WFH on the work experience, the work experience measure (WEM), with higher values implying more positive impact. Computations were carried out using the R packages psych (24) and lavaan (25).

The univariate association of sociodemographic, individual, familiar and organizational factors with WEM was analyzed by Wilcoxon test and Kruskal-Wallis test. The WEM score was recoded into three classes of increasingly positive impact using the tertiles of its sample distribution. A proportional odds model for the categorical WEM response was used to determine adjusted odds ratios and 95% CIs. The AIC and the likelihood ratio test were used to identify the final model. The assumption of proportional odds was checked by the likelihood ratio test comparing the multinomial model to the main proportional odds model (26, 27).

The level of significance was fixed at 5%. Unless otherwise specified, significant association will be a short for statistically significant association. Statistical analysis was performed by R (28).

## 3. Results

### 3.1. Sample characteristics

A total of 748 participants (median age from 50 to 59 years) completed the questionnaire and, after validation of the data, all respondents have been included in the study. A total of 733 subjects (98%) completed all the four sections (742 section 2, 737 section 3, 740 section 4). The completion rates ranged from 78.4 to 79.6%. A flow chart about study participants is presented in Supplementary Figure 1.

Women represent 57.6% of respondents, including <1% who chose “Other” or “I prefer not to answer.” General characteristics of the sample and the CNR population are reported in Table 1. According to gender and geographic distribution, women and employees living in the North of Italy were over-represented in the sample. Concerning age, there was a slight under-representation of both younger and older employees. A higher percentage of researcher and technologists with respect to the administrative and technical staff participated to the survey.

**Table 1:** Participants' general characteristics. Comparison with the source population as of December 2021, 31st, excluding managers, where feasible. Source CNR.

	**Source population n = 8**, **543**	**Sample** **n** = **748**	
	**%**	***N***	**%**
**Gender**			
Man	53.0	317	42.4
Woman	47.0	431	57.6
**Age group (years)**			
≤ 39	14.9	90	12.0
40–49	35.1	275	36.8
50–59	35.3	285	38.1
≥60	14.7	98	13.1
**Living status**			
Living alone	–	108	14.4
Married or living together, no children	–	282	37.7
Married or living together, with children	–	358	47.9
**Italian macro-region**			
North	24.0	244	32.6
Center	40.4	261	34.9
South	25.1	168	22.5
Islands	10.5	75	10.0
**Education level**			
Bachelor's degree or higher	–	614	82.1
Less than a bachelor's degree	–	134	17.9
**Professional profile**			
Administrative and technical staff	38.2	238	31.8
Researcher and technologist	61.8	510	68.2

### 3.2. Main perceived changes in the life domain

More than 95% of the respondents reported a perceived change in at least one item of the life domain. Approximately 30% of respondents positively and 6.3% negatively assessed the impact on lifestyle, sleep quality and psychological status (Q1–Q3) simultaneously. The 17% of subjects responded positively to all aspects of the life domain, while 1.6% negatively.

**Figure 1:**
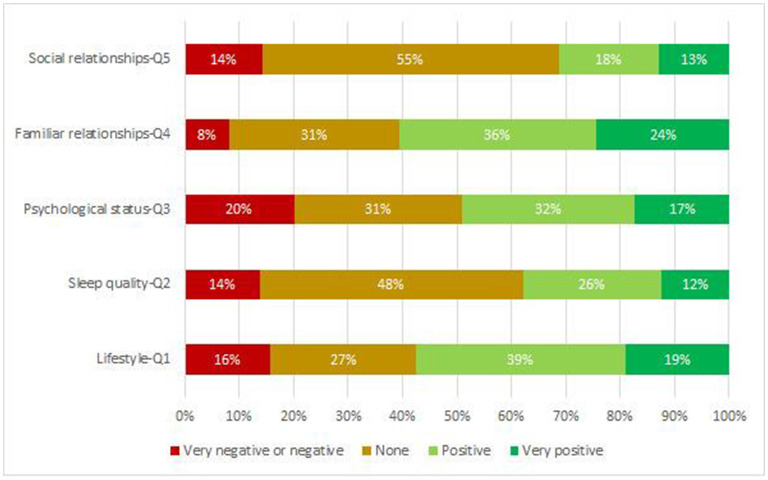
Perception of the impact of WFH on: Q1- lifestyle, eating habits and state of health; Q2-quantity/quality and disturbances of sleep, and daytime sleepiness; Q3-psychological status; Q4-quality of interpersonal relationships within the family; Q5-quality of interpersonal relationships within the network of friends. Very negative and negative rates have been combined due to very low frequencies of the very negative rate.

Figure 1 shows the frequency distributions of self-reported perceptions of the impact on the life domain of WFH. For the sake of clearness, very negative and negative rates were combined due to low frequencies of the very negative responses (<1.5%). Aside from a large group of subjects reporting that WFH had no impact (27 to 55%), there was a clearly prevalent positive vote (30 to 60%). The lack of any impact was highly prevalent with respect to sleep disturbances quality (Q2, 48%) and relationships within the network of friends (Q5, 55%). The negative evaluation of the impact appears more frequently in relation to the psychological status (Q3, 20%) while the positive evaluation was given more frequently in relation to the quality of interpersonal relationships within the family (Q4, 60%) and lifestyle, eating habits and health status (Q1, 58%). In the latter two cases, the percentage of very positive responses was higher than the percentage of negative ones.

A strong consistency was found between self-reported perceived impacts and variations in MeDAS, PSQI, ESS and PHQ total scores. Significant association was found for each assessment (see Supplementary Table 1) and in each case all the relevant standardized residuals were positive and significant (*p* < 0.001). The increase in adherence to the Mediterranean diet as measured by the MeDAS score was significantly higher within the subjects rating positively on (Q1) than within the other two groups of subjects (adjusted *p* < 0.0002). As far as PSQI, ESS and PHQ are concerned, significant decreasing trends of the median variations along with increasingly positive perception of the impact of WFH on (Q2) and (Q3) were obtained, and all the pairwise comparisons were statistically significant (see Supplementary Figure 2).

### 3.3. Main perceived changes in the work domain

**Figure 2:**
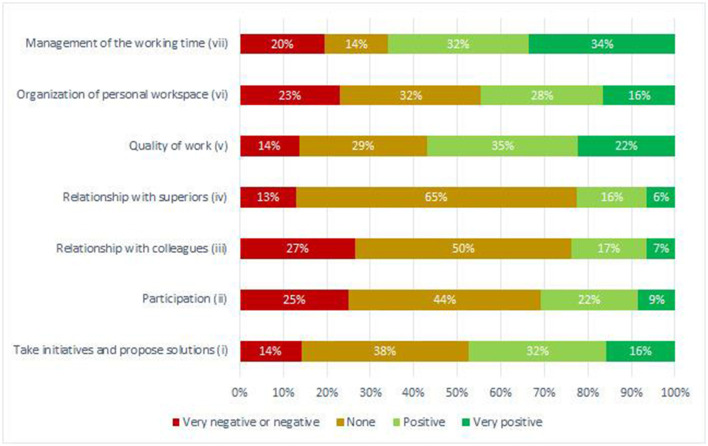
Perception of the impact of WFH on: (i) ability to take initiatives and propose solutions in the workplace; (ii) participation in the working context; (iii) relationship with colleagues; (iv) relationship with superiors; (v) quality of work; (vi) organization of personal environment and workspace; (vii) management of the working time. Very negative and negative rates have been combined due to very low frequencies (≤3%) of the very negative rate.

Overall, over 97% of respondents reported a perceived change in at least one job dimension: 12% rated the impact on all job-related items positively while <2% rated the experience as completely negative. Figure 2 shows in more detail how WFH was perceived to influence the work experience. For the sake of clearness, very negative and negative rates were combined due to low frequencies of the very negative responses (≤3%). The participation and relational aspects are those in which the perception of absence of impact prevailed (44–65%). But, at the same time, the items on the relationship with colleagues and the participation in the work context collected the greatest number of negative responses (27 and 25%, respectively). It stands out that negative perceptions (27%) prevailed over positive ones (24%) in the subject of relationships with colleagues. Positive perceptions prevailed over both negative perceptions and lack of impact perceptions in the subjects of flexibility (organization of personal workspace and management of the working time), taking initiatives and proposing solutions, and quality of work.

**Figure 3:**
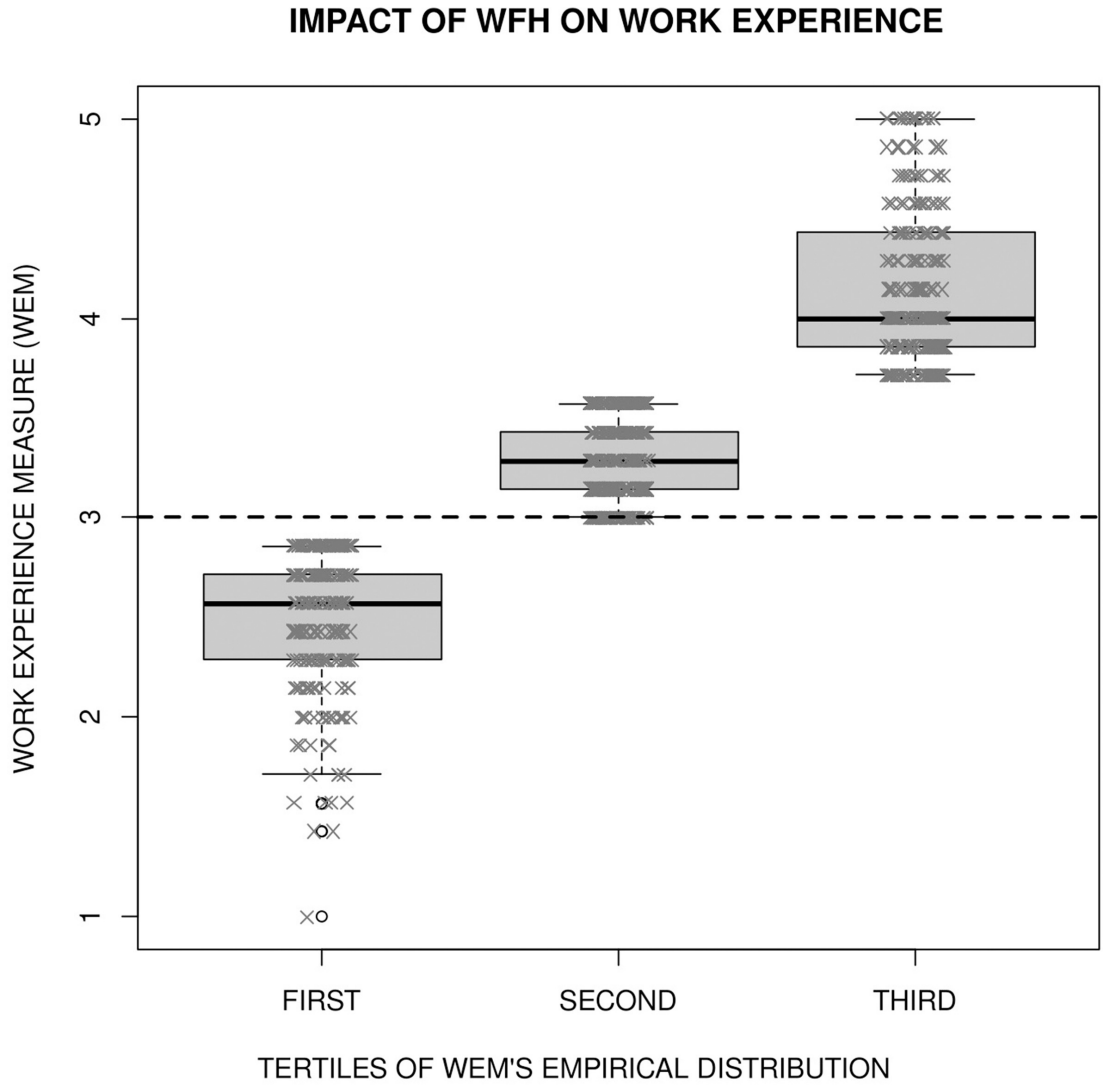
Empirical distribution of the WEM rating the impact of WFH on the work experience in the three classes defined by the tertiles 3 and 3.71.

Most of the subjects (64%) obtained a value of the work experience measure >3 (mean = 3.362, s.d.= 0.746, median = 3.357, interquartile range from 2.86 to 3.86), and all the range of the 5-point Likert-type scale from 1 (very negative) to 5 (very positive) was used. Figure 3 shows that the three classes defined by the tertiles 3 and 3.71 of the WEM's empirical distribution can be reasonably interpreted as a negative (WEM <3), moderately positive (3 ≤ WEM < 3.71) and very positive (WEM ≥ 3.71) perceived impact of WFH on the working experience.

### 3.4. Factors associated with the perceived changes in the life and work domains: bivariate analysis

Table 2 reports the bivariate associations of life domain (Q1-Q5) and work domain (WEM) with demographics, individual, family and organizational characteristics. As a small percentage of negative responses has been recorded in Q4 (see Figure 1), from Q4 and Q5 a new variable Q4/5 was defined considering only the worst response of the two, so as to better balance the groups in a conservative way. Since there were no significant differences in perceived impacts between men and women (*p* > 0.12), the analysis in this section was not stratified by gender. All the remaining factors had at least a weak association with at least one of the considered outcomes. The following factors showed a significant association with all the outcomes: frequency of sharing the work room at home (*p* ≤ 0.03), time to get from home to work (*p* ≤ 0.006), number of days of work in presence (*p* ≤ 0.02), sedentary lifestyle (*p* < 0.001), vigorous physical activity (*p* ≤ 0.006), moderate physical activity (*p* ≤ 0.006), hobbies/pastimes (*p* ≤ 0.002) and more weakly, size of the city of residence (*p* ≤ 0.10).

**Table 2:** Bivariate association of socio-demographic, individual, familiar and individual organizational factors with life dimension and work dimension.

	***p*** **-value in the bivariate analysis**				
**Factors**	**Q1**^a^ **(lifestyle and health)**	**Q2**^a^ **(sleep quality)**	**Q3**^a^ **(psychological status)**	**Q4/5**^a^ **(family and friends)**	**WEM**^b^ **(work experience)**
Gender	0.80	0.37	0.12	0.33	0.95
Age group	<0.001^§^	<0.001^§^	0.14	0.02^§^	0.32
Living alone	0.27	0.009^§^	<0.001^§^	0.36	0.32
Children at home for >6 months	0.46	0.08	0.32	0.57	0.80
Macro-region of residence	0.13	0.19	0.20	0.01	0.11^§^
**Size of the city of residence^c^**	0.04^§^	0.01	0.02	0.10	0.008
Size of the house	0.11	0.13	0.04	0.05	0.03
Availability of a fixed workstation at home	0.75	0.08	0.15^§^	0.001^§^	<0.001^§^
**Frequency of sharing the work room at home**	0.03^§^	0.02^§^	0.01^§^	0.002^§^	0.002^§^
Assistance to cohabitants	0.17	0.02	0.04^§^	0.45	0.26
Assistance to non-cohabitants	0.63	0.41	0.11	0.09	0.02^§^
**Time to get from home to work**	<0.001^§^	<0.001^§^	0.001^§^	0.006^§^	<0.001^§^
**Number of days of work in presence**	0.001^§^	0.005	0.02	0.02	<0.001^§^
Graduation	0.13	0.13	0.02^§^	0.06	0.03
Professional profile	0.12	<0.001^§^	0.01	0.12^§^	0.002^§^
**Sedentary lifestyle**	<0.001^§^	<0.001^§^	<0.001^§^	<0.001^§^	<0.001^§^
**Vigorous physical activity**	<0.001^§^	<0.001	0.003	<0.001	0.006
**Moderate physical activity**	0.006^§^	0.01	0.02^§^	0.004	0.002
Habit of walking	0.02	0.23	0.44	0.62	0.62
**Hobbies/pastimes**	<0.001^§^	<0.001^§^	<0.001^§^	0.002^§^	0.002

a Univariable multinomial logistic regression.

b Either Nonparametric ANOVA or Wilcoxon test.

c The bold character highlights variables associated with all the indicated items based on *p* ≤ 0.10.

§ Variables included in the final multivariable model for each item.

All these common factors showed the same relationship with each of the self-reported outcomes. Subjects living in larger cities were more likely to rate negatively all the item of the life domain. Moreover, the WEM was significantly lower among subjects living in the largest cities (> 150000 habitants) with respect to those living in the smaller ones (< 50000 habitants). As far as home-to-work travel time is concerned, among the subjects with a longer time (> 15 minutes) the WEM value was significantly higher. Living away from the workplace made it more likely a positive judgment in every aspect of the life domain as well. In our sample, home-to-work travel time was strongly associated with the size of the city of residence (*p* < 0.0001), with a significant number of subjects living in small cities far from the workplace and, vice versa, with a significant number of individuals living in larger cities near the workplace. Subjects who were able to make fewer days of work in presence were more likely to rate all the items of the life domain positively and to have a higher WEM value. Individuals who had to frequently share the work room with other family members were more likely to rate all the items of the life domain negatively and to have a WEM value significantly lower than those who never shared. A high frequency of sharing the work room at home was not associated with gender (*p* = 0.19).

Diverse habits with respect to individual factors such as pursuing hobbies or physical activity were also associated with a different perception of the impact of WFH on both the life and work domains. Respondents who had no hobbies or pastimes prior to the pandemic but started cultivating them in the WFH period were more likely to give a positive score to all but Q1 items of the life domain and scored higher on the WEM scale. Moreover, subjects who previously had hobbies or pastimes and then abandoned them were more likely to give a negative response. The median WEM value in this latter group was < 3, toward a negative assessment of WFH impact on the work experience, and was significantly lower than the median value in both the groups of subjects having any hobbies/pastimes during WFH (both new and pre-pandemic). The group that abandoned hobbies and pastimes, however, was the smallest group (8.2%) almost equivalent to that of individuals who started to be engaged in hobbies or pastimes (8.6%). In fact, 71% of respondents participated in hobbies or pastimes before the pandemic and also during the WFH period. In our sample, significantly fewer women than men were able to maintain previous hobbies and pastimes during the WFH period.

As far as moderate or vigorous physical activities are concerned, the main results were quite similar to each other and, in principle, similar to those obtained for hobbies and pastimes. In our sample, the 35% of respondents were regularly exercising in either moderate or vigorous physical activity during the WFH period. Among the subjects regularly practicing moderate physical activity (58%), more than half increased while about 15% decreased the time spent on training with respect to the pre-pandemic period. Stopping physical activity due to COVID19 restrictions significantly increased the odds of a negative impact of WFH on the life domain. WEM also was significantly lower among those who stopped exercising than among those who regularly were practicing. In turn, subjects who maintained the physical activity but decreased the time devoted to it, scored significantly lower than those who increased the time, even much. Analogously, decreased time spent walking was associated with the lowest WEM score, while an increased time with the highest score. All these results are consistent with the significant association between a reduction in sedentary life and a higher WEM value and, conversely, between an increase in sedentary lifestyle and a lower value. Subjects who have reduced their sedentary lifestyle were more likely to rate all the life domain items positively. On the contrary, those who much increased their sedentary lifestyle were more likely to rate the impact of WFH on their family and friendship relationships negatively, and their WEM value was significantly lower (median <3).

Women more than men significantly decreased their sedentary lifestyle (*p* = 0.005). Among the respondents who did not change their sedentary lifestyle, significantly more than expected maintained the habit of hobbies and pastimes and conversely, significantly less than expected abandoned this habit. Abandoning hobbies and pastimes was significantly more frequent among the subjects who increased their sedentary life style.

### 3.5. Factors associated with changes in the items of the life domain: multivariable regression analysis

To determine effect measures in the form of adjusted odds ratios, multivariable multinomial logistic regression models were applied to the three classes of Q1-Q3 and Q4/5 outcomes (see section 2.4.1). The no perceived impact group was considered as the reference group. The final multivariable models met the non-collinearity requirement based on the conservative vif threshold of 5. The summary of the obtained results is presented in Supplementary Table 2. When compared to subjects who reported a lack of impact of WFH, respondents who lived not very close to their office, those who reduced their sedentary lifestyle and those who started to be engaged in hobbies or pastimes during the WFH period, have been more likely to rate the impact of WFH on all the aspects of life and work domains positively.

When adjusting for other covariates, subjects who regularly exercised were more likely to rate the impact of WFH on their psychological status (Q3) positively. A trend in the same direction was obtained for the impact on lifestyle (Q1). Stopping to pursue hobbies or pastimes during the WFH period made 5 times more likely a rate of negative impact on Q3. However, compared to the group of respondents with no habit of hobbies and pastimes, those who stopped their activities were about 4 times more likely to rate the WFH's impact on Q3 positively.

Lack of changes in the sedentary lifestyle significantly prevented a positive rating on all the life domain dimensions. Furthermore, subjects who much increased their sedentary lifestyle were 2.6 times more at risk than subjects who decreased of negatively evaluating the impact on the quality of interpersonal relationships within the family and the network of friends.

Respondents who lived alone during the WFH were more likely to rate the impact on their quality/quantity of sleep and psychological status negatively. Considering also weak significances, a frequent sharing of the work room had a negative impact on all the aspects of the life and work domains (see also the following sub-section). Subjects who had the highest number of office days during WFH were less likely to rate the impact on lifestyle, eating habits and state of health positively.

### 3.6. Factors associated with changes in the perception of the work experience: multivariable regression analysis

In order to disentangle specific individual, familiar and individual organizational factors influencing the way work experience was perceived, Q1-Q5, as summary indices, were excluded from the logistic analysis of WEM determinants despite the significant association of all of them with WEM. In slightly more detail, a significantly increasing median value of WEM along the three classes of negative, none and positive impact of WFH on Q1-Q3 and Q4/5 was found (*p* = 0; all the pairwise comparisons were significant, *p* = 0).

According to the estimated proportional odds logistic model, the availability of a work room (OR: 1.56, 95%CI: 1.12-2.18), any time > 15 minute to go from home to the office (ORs: from 1.87 to 3.96), being a member of the administrative staff compared to being a researcher (OR: 2.32, 95%CI: 1.42-3.88), the need to assist a non-cohabitant person (OR: 1.36, 95%CI: 1.01-1.85) and living on one of the major Islands compared to live in the North of Italy (OR: 2.04, 95%CI: 1.23-3.41) were significantly associated with a more positive assessment of the WFH impact on work experience. On the other hand, subjects who increased their sedentary lifestyle (ORs from 0.26 to 0.40), those who had to often share the work room (OR: 0.56, 95%CI: 0.39-0.81) and those who had the highest number of office days during WFH (OR: 0.62, 95%CI: 0.40-0.93) were more likely to rate the impact of WFH negatively. See Supplementary Table 3 for a complete list of results, where the reported ORs refer to the outcomes high WEM versus a lower WEM, according to the R's parameterization of the model.

## 4. Discussion

Albeit with some distinctions, working from home has been extended in the Italian Public Administration for about 18 months after the beginning of the pandemic. Unlike other studies that focused on lockdown periods, we investigated how the whole experience of working from home has affected a few aspects that define the perceived quality of life (29) such as lifestyle and health status, quality of sleep and psychological status, quality of interpersonal relationships within both the family and the network of friends, and work experience in a community of knowledge workers. Research workers in particular, as they act mainly on non-material processes, apply subjective judgment to tasks and have large autonomy in organizing their work, should ideally have been better prepared than other categories of workers to deal with remote work. Despite this, not even the world of academic research seems to derive full benefits from smart working. Although less studied than other work environments, negative effects such as isolation, loss of feedback and collegial reinforcement, inadequate communication, lack of opportunities for skills development and even lower work efficiency have been highlighted in this environment as well (30, 31).

In our sample, the results on the life domain were characterized by the prevalence of a self-reported positive or very positive perception. The CNR does not have family support tools other than economic subsidies, such as company kindergartens or after-school facilities, except at an isolated and local level. Tools to promote individual well-being are also lacking. Considering also the rigidity of work organization before the pandemic, the prolongation of WFH seems to have made up for the lack of adequate policies, as well as playing the primary role of preventing contagion. It is interesting to note how our result differs from the survey by Cellini and colleagues (14), which showed that in spring 2020, 80% of CNR respondents did not consider the possibility to work from home as one of the main positive aspects of the WFH. However, to the question “Do you think having worked in smart working in exceptional conditions may have influenced your perception of smart working?” 39% of that sample answered Probably yes and 27% Certainly yes. The comparison, albeit indirect, between the two studies supports our assumption that by analyzing perceptions over the medium term, we can obtain a different picture than what is reported by the literature referring to lockdown periods only. This is also testified by the fact that currently just under 75% of staff have signed an individual agile working agreement against 54% of CNR respondents from the study by Cellini and colleagues who in spring 2020 reported are planning to apply for an extension of the smart-working at the end of the pandemic (probably yes, 32%; surely yes, 22%).

Except for gender, nearly all socio-demographic, individual, family and organizational factors were associated in bivariate analyses with the reported perceptions. As highlighted in Section 3, a few factors were associated with all the items investigating the life domain, also after adjustment for other covariates. The four most important factors were: the time taken to get from home to work (socio-demographic factor), the frequency of sharing the work room at home (individual organizational factor), changes in hobbies/pastimes and in sedentary habits (individual factors).

Reducing travel times is a well-known beneficial effect of smart working because, in addition to reducing costs, it allows for better management of work and family time, and the availability of more free-time. In our sample, 50% of respondents took more than 30 minutes to go from home to work, 17.5% at least 60 minutes and only 30% used public transport or walked. In general, to translate these positive aspects for Italian employees into reality, it is necessary that there should be investments in technological infrastructures especially in the smaller urban centers (Italy ranked 24th in DESI 2019^1^). In (14) it has been show that about 1 in 5 CNR worker in the sample complained about too slow connections, overloading of lines which prevented continuity of work and inability to remotely access own pc in the office. Furthermore, it is necessary that the time not spent in commuting does not translate entirely into additional working time, negatively affecting the work-life balance. The fact that part of the saved time may go directly back to the employer in the form of additional work time has already been proved a benefit to employers (10, 14, 32).

Among the individual organizational factors, a high frequency of work room sharing is associated with a perceived negative impact on all life domain issues, especially psychological status. The frequency of sharing was significantly associated with the presence of children in the house for at least 6 months during the WFH period, and with the number of children. Italy indeed is one of the European countries where schools were closed for the longest time (33). As expected, subjects with two or more school-age children (then experiencing remote learning and/or daycare closures, 25.3%) had to share the work room more often than others.

The important role played by leisure time activities on health and quality of life (34, 35) even more during the pandemic (36–38) is well-known. In our survey, we took into account changes in habits and frequency of physical activity, hobbies/pastimes and sedentary lifestyle in general. In the sample, be engaged in hobbies/pastimes and exercising were two important explaining factors of the positive impact on the life domain of WFH. Increased sedentary lifestyle was reported during COVID-19 lockdowns (37) due to social distancing and isolation policies. In our sample, almost half of the subjects (48%) reported increased sedentary lifestyle over a much longer period. As for health, institutional training at CNR focuses mainly on job security and during the WFH period, on working from home safety standards. Our results suggest the need for continuous training policies that promote physical and mental health by countering the tendency to sedentary lifestyles (39, 40) even with specific programs, as recommended during COVID-19-related restrictions on physical activity (41).

Changes in sedentary lifestyle were significantly associated with gender: in our sample women more than men significantly decreased their sedentary lifestyle. However, significantly fewer women than men were able to maintain previous hobbies and pastimes during the WFH period. Moreover, less women than men were used to vigorous or moderate physical activity. It would seem, therefore, that working from home has meant that women devoted more time to home and family. Although many studies have shown that the family burden increased for women who had to work from home, with lower satisfaction with their work-life balance (42, 43), in our sample we did not find gender differences in the reported perceptions. The frequency of sharing the work room, which can be considered one of the evident elements of the overlap between work and family life, was not associated with gender. Although not directly addressed in this study, this lack of gender difference could be due to the increased involvement of fathers in childcare and homeschooling activities highlighted by several studies conducted during the pandemic (42, 44). After the experience lived during the WFH period, the maintenance on a large scale of flexible forms of work without their use penalizing the worker's career could in general favor greater adherence to company policies for the reduction of family conflicts, commonly underused by fathers even in organizations where these policies are more developed (45). These elements must be taken into account in planning the CNR's gender equality strategy. As gender differences can be masked in our study by other considered factors, it is necessary to better understand whether the increased flexibility in work corresponds to a real improvement in gender equality. Women could indeed gain more, from both the flexibility in their work and a more balanced distribution of family burdens (46). This could have a positive impact on children's wellbeing as well (47).

Although to a slightly lesser extent, the impact on work experience was also positively assessed. Apart from the selection bias issue, the overall positive impact on both the domains could also be an effect of the longer perspective of the study, as any perceived deterioration in quality of life and work experience due to the exceptional initial conditions has probably already been overcome. Similarly, the difficulties associated with low digital skills should have been overcome by now. The 7 items of the work domain investigated some well-known critical issues of WFH such as coordination and cooperation among workers (i-ii), relationship with the management (iv), work isolation (iii) and flexibility (vi-vii) which can interact with the work-life balance (8). The fact that relationships with superior and colleagues and participation in the work context were the items with the highest frequency of negative responses testifies to isolation as an aspect of concern for smart working. This was already noted in the relevant literature (48) but cannot be taken for granted as it is strongly related to the attitude of the single individual (49). The aspect of isolation also emerged in the study of Cellini and colleagues, but was attributed to the exceptional conditions of the beginning of the pandemic. In our study this aspect is confirmed as of general relevance. In our sample, researchers significantly less often than others reported a positive impact on participation in the work context and on relationships with colleagues. The perceived isolation can negatively affect knowledge sharing, future cooperation and overall work engagement (50, 51). To prevent this, policies aimed at strengthening inclusion should be implemented. During the pandemic period, the CNR organized online training sessions on the general topics of COVID19, best protection practices and vaccination. This activity also had the objective of reducing isolation by sharing the expert knowledge of colleagues on a topic of general interest. Scheduling similar activities on a regular basis can help maintain a sense of community. In addition, specialized training should be conducted for leaders focusing on how to support the workforce, connect employees and strengthen a sense of belonging to the CNR community. Until now, in fact, training for executives has mainly focused on regulatory aspects. Work engagement is negatively correlated with burnout (52), a specific phenomenon of the occupational context traditionally studied among health care workers and helping professions (53) and widely studied also among teachers, professors and academic staff since the end of the last century (54, 55). Emotional exhaustion is generally considered the core of the burnout concept, as a response to excessive work demands that run out the worker's emotional resources (56). In our sample, the work experience measure was positively correlated with all the items of the life domain suggesting a higher risk of burnout for subjects rating negatively the impact of WFH on life or work domain. A frequent sharing of the work room is one of the factors negatively affecting the work experience as well as the life domain in our sample, and one of the factors affecting emotional exhaustion in academic staff (57). Other related factors intervened on the perception of work experience. In fact, the availability of a room in which to work permanently favored a positive evaluation, being associated in our sample with a less frequent sharing of the work space with other family members. When a worker has to choose between ordinary ways of working and a flexible form of work, these family and individual organizational aspects must not be underestimated.

This study has several limitations, first of all the self-reported nature of the perceived impact of WFH. Second, as in several studies conducted at the beginning of the pandemic (e.g., (58, 59)) or even later (60), we asked colleagues to report pre-pandemic habits and sensations and therefore our study is also subject to the problem of recall bias. Retrospective questionnaires can be useful when other studies are not feasible, but only a few studies have validated the use of retrospective questionnaires in specific situations, (e.g. (61, 62)). In this study, the fact that recall bias may have resulted in removing initial negative effects due to the health crisis more than working from home was, in reality, an intended effect. However, the long period of time that has passed since the outbreak may have affected the memory of previous everyday life and this may have led to the prevalence of the no-impact option. Third, the study population consisted of a self-selected group of employees. Subjects who positively perceived the period of forced WFH both personally and at work, may have had greater motivation to join the survey. This can partly explain the lower participation of technical and administrative staff, whose tasks can only partially be performed remotely. Fourth, the response rate was only around 10%. From the point of view of the employee-employer relationship, it cannot be excluded that the low participation in the survey was also caused by a perception of employer control despite all the guarantees that the research group tried to give in terms of privacy and purposes of the investigation, as described in section 2.3. Since the survey was launched immediately after the regulation of smart working by the CNR (BoD resolution no. 203 of 21 December 2021) we nevertheless believe that the compilation was not influenced by fears or expectations regarding impacts of the survey on organizational prospects. Fifth, we administered the survey in a single research center, and therefore some results may depend on the specific organizational framework and therefore, cannot be generalized tout court to other knowledge worker communities nor, least of all, to other sectors of the Italian public administration. Sixth, the use of an *ad-hoc* questionnaire does not allow comparison with other similar studies. There is also the possibility that some factors of interest were not included in the survey. Income, for example, has not been included for privacy reasons, however age and occupational profile can be considered together as an approximation of earned income. Furthermore, given the length of the questionnaire, we were unable to investigate in detail the aspects related to physical activity and hobbies/pastimes. Finally, the impact on the life domain was explored with single item questions rather than by using a validated quality of life questionnaire. The work experience measure developed in this study may not have fully captured the most critical issues to work activity. The life and work dimension measures were highly associated each other, which may explain the presence of almost the same predictors across the models.

The study has also important strengths. First, almost all the respondents completed the questionnaire in full, making it possible to verify in depth the consistency of the answers given. Second, the ability to confirm the self-reported behaviors on lifestyle, sleep quality and psychological status with objective measures based on the clinical questionnaires.

The results presented are in line with expectations, with some exceptions such as lack of gender differences. However, the fact that some factors together drive all the reported perceptions lends some strength to our results. Therefore, this study can provide general suggestions for working contexts in which adequate company policies for work-life balance and support for smart workers' wellbeing are still lacking.

## Data availability statement

The original contributions presented in the study are included in the article/Supplementary material, further inquiries can be directed to the corresponding author.

## Author contributions

SG and ES selected validated questionnaires. AB, CL, PM, GP, AR, and SS developed the section investigating socio-demographic, individual, and family and individual organizational factors. MT and SS followed the entire ethical approval process. RG and SS took care of the online implementation aspects of the survey. GP ideated the 7-item scale for work dimension. AB and GP carried out items analysis on the collected data and collaborated on the preliminary, general analyses. AB, CL, and AR conceived the work and interpreted data and inferential results. AB carried out statistical analyses and wrote the original draft of the manuscript. All authors conceived the survey, critically reviewed the manuscript, read and approved the final version of the manuscript.

## Footnotes

^1^ https://digital-strategy.ec.europa.eu/en/library/digital-economy-and-society-index-desi-2019

## References

[1] Riccardo F Ajelli M Andrianou XD Bella A Del Manso M Fabiani M. Epidemiological characteristics of COVID-19 cases and estimates of the reproductive numbers 1 month into the epidemic, Italy. Eur Commun Dis Bull. (2020) 25:2. 10.2807/1560-7917.ES.2020.25.49.2000790 33303064 PMC7730489

[2] ISTAT ISTAT The organization of work in Italy: times places degree degree of autonomy. (2020) (in Italian). Press release. Available online at: https://www.istat.it/it/archivio/247627 (accessed January 24, 2023).

[3] ISTAT Censimento permanente delle Istituzioni pubbliche: risultati preliminari. (2020). l'anno dello Smart Working; 2021 (in Italian). Press release. Available online at https://www.istat.it/it/archivio/264696 (accessed January 24, 2023).

[4] Milasi S González-Vázquez I Fernández-Macías E. Telework in the EU before and after the COVID-19: where we were, where we head to. European Union - JRC 120945. (2021). Available online at: https://joint-research-centre.ec.europa.eu/system/files/2021-06/jrc120945_policy_brief_-_covid_and_telework_final.pdf (accessed January 24, 2023).

[5] Ripamonti SC Galuppo L Provasoli G Benozzo A. Unmasking reflexivity in HR managers during the COVID-19 lockdown in Italy. Front Psychol. (2020) 11:588128. 10.3389/fpsyg.2020.588128 33424703 PMC7786014

[6] Vyas L Butakhieo N. The impact of working from home during COVID-19 on work and life domains: an exploratory study on Hong Kong. Policy Des. Pract. (2021) 4:59–76. 10.1080/25741292.2020.1863560

[7] Beckel JLO Fisher GG. Telework and worker health and well-being: a review and recommendations for research and practice. Int J Environ Res Public Health. (2022) 19:3879. 10.3390/ijerph19073879 35409563 PMC8998114

[8] Bolisani E Scarso E Ipsen C Kirchner K Hansen JP. Working from home during COVID-19 pandemic: lessons learned and issues. Manag. Mark Challenges Knowl Soc. (2020) 15:458–76. 10.2478/mmcks-2020-002733071425

[9] McDowell CP Herring MP Lansing J Brower C Meyer J.D. Working from home and job loss due to the COVID-19 pandemic are associated with greater time in sedentary behaviors. Front Public Heal. (2020) 8:597619. 10.3389/fpubh.2020.597619 33224922 PMC7674395

[10] Tejero LMS Seva RR Fadrilan-Camacho VFF. Factors associated with work-life balance and productivity before and during work from home. J Occup Environ Med. (2021) 63:1065–72. 10.1097/JOM.0000000000002377 34560760 PMC8630924

[11] Weitzer J Papantoniou K Seidel S Klösch G Caniglia G Laubichler M. Working from home, quality of life, and perceived productivity during the first 50-day COVID-19 mitigation measures in Austria: a cross-sectional study. Int Arch Occup Environ Health. (2021) 94:1823–37. 10.1007/s00420-021-01692-0 33877416 PMC8056371

[12] Ipsen C Kirchner K Hansen JP. Experiences of working from home in times of covid-19 International survey conducted the first months of the national lockdowns March-May 2020. DTU Manag. (2020) 3:1–32. 10.11581/dtu:00000085

[13] Birimoglu Okuyan C Begen MA. Working from home during the COVID-19 pandemic, its effects on health, and recommendations: the pandemic and beyond. Perspect Psychiatr Care. (2022) 58:173–9. 10.1111/ppc.12847 34003489 PMC8242705

[14] Cellini M Pisacane L Crescimbene M Di Felice F. Exploring employee perceptions towards smart working during the COVID-19 pandemic: a comparative analysis of two Italian public research organizations. Public Orga Rev. (2021) 21:815–33. 10.1007/s11115-021-00559-9

[15] Biron M Peretz H Turgeman-Lupo K. Trait optimism and work from home adjustment in the COVID-19 pandemic: considering the mediating role of situational optimism and the moderating role of cultural optimism. Sustainability. (2020) 12:9773. 10.3390/su12229773

[16] Mannocci A Masala D Mei D Tribuzio AM Villari P La Torre G. International physical activity questionnaire for adolescents (IPAQ A): reliability of an Italian version. Minerva Pediatr. (2021) 73:383–90. 10.23736/S2724-5276.16.04727-7 29381006

[17] García-Conesa MT Philippou E Pafilas C Massaro M Quarta S Andrade V. Exploring the validity of the 14-item mediterranean diet adherence screener (MEDAS): a cross-national study in seven European countries around the mediterranean region. Nutrients. (2020) 12:2960. 10.3390/nu12102960 32992649 PMC7601687

[18] Curcio G Tempesta D Scarlata S Marzano C Moroni F Rossini PM. Validity of the Italian version of the pittsburgh sleep quality index (PSQI). Neurol Sci Off J. (2013) 34:511–9. 10.1007/s10072-012-1085-y 22526760

[19] Vignatelli L Plazzi G Barbato A Ferini-Strambi L Manni R Pompei FD'Alessandro R GINSEN (Gruppo Italiano narcolessia studio epidemiologico nazionale). Italian version of the Epworth sleepiness scale: external validity. Ital J Neuro. Sci. (2003) 23:6. 10.1007/s100720300004 12624716

[20] Natale V Esposito MJ Martoni M Fabbri M. Validity of the reduced version of the morningness–Eveningness questionnaire. Sleep Biol Rhythms. (2006) 4:72–4. 10.1111/j.1479-8425.2006.00192.x

[21] Kroenke K Spitzer RL Williams JB. The PHQ-9: validity of a brief depression severity measure. J Gen Intern Med. (2001) 16:606–13. 10.1046/j.1525-1497.2001.016009606.x 11556941 PMC1495268

[22] Mazzotti E Fassone G Picardi A Sagoni E Ramieri L Lega I. The Patient Health Questionnaire (PHQ) for the screening of psychiatric disorders: a validation study versus the Structured Clinical Interview for DSM-IV axis I (SCID-I). J Psychopathol. (2003) 3:19.

[23] Johnston R Jones K Manley D. Confounding and collinearity in regression analysis: a cautionary tale and an alternative procedure, illustrated by studies of British voting behaviour. Qual Quant. (2018) 52:1957–76. 10.1007/s11135-017-0584-6 29937587 PMC5993839

[24] Revelle W. Procedures for Psychological, Psychometric, and Personality Research 2022.

[25] Rosseel Y. lavaan: An R package for structural equation modeling. J Stat Softw. (2012) 48:1–36. 10.18637/jss.v048.i0225601849

[26] Hosmer DW Lemeshow S. Applied Logistic Regression, Second Edition. New York, NY: John Wiley & Sons, Inc. (2000).

[27] Agresti A. An Introduction to Categorical Data Analysis, Second Edition. Hoboken, NJ: John Wiley & Sons, Inc. (2006).

[28] R Core Team. R: A Language Environment for Statistical Computing. Vienna: R Foundation for Statistical Computing (2022). Available online at: https://www.R-project.org/

[29] WHO Team Mental Health and Substance Use. The World Health Organization Quality of Life (WHOQOL). Geneva: World Health Organization (2012) WHO/HIS/HSI Rev.2012.03.

[30] Aczel B Kovacs M van der Lippe T Szaszi B. Researchers working from home: Benefits and challenges. PLoS ONE. (2021) 16:e0249127. 10.1371/journal.pone.0249127 33765047 PMC7993618

[31] Adisa TA Antonacopoulou E Beauregard TA Dickmann M Adekoya OD. Exploring the impact of COVID-19 on employees' boundary management and work–life balance. Br J Manag. (2022) 33:1694–709. 10.1111/1467-8551.12643

[32] Sousa-Uva M Sousa-Uva A e Sampayo MM. Telework during the COVID-19 epidemic in Portugal and determinants of job satisfaction: a cross-sectional study. BMC Public Health. (2021) 21:2217. 10.1186/s12889-021-12295-2 34865641 PMC8645416

[33] UNESCO Global School Closures COVID-19. (2022). Available online at: https://data.humdata.org/dataset/global-school-closures-covid19 (accessed January 23, 2023).

[34] Vuillemin A Boini S Bertrais S Tessier S Oppert JM Hercberg S. Leisure time physical activity and health-related quality of life. Prev Med. (2005) 41:562–9. 10.1016/j.ypmed.2005.01.006 15917053

[35] Cunningham CO' Sullivan R Caserotti P Tull MA. Consequences of physical inactivity in older adults: a systematic review of reviews and meta-analyses. Scand. J Med Sci Sports. (2020) 30:816–827. 10.1111/sms.13616 32020713

[36] Haider S Smith L Markovic L Schuch FB Sadarangani KP Lopez Sanchez GF. Associations between physical activity, sitting time, and time spent outdoors with mental health during the first COVID-19 lock down in Austria. Int J Environ Res Public Health. (2021) 3:8. 10.3390/ijerph18179168 34501758 PMC8431505

[37] Karageorghis CI Bird JM Hutchinson JC Hamer M Delevoye-Turrell YN Guérin SMR. Physical activity and mental well-being under COVID-19 lockdown: a cross-sectional multination study. BMC Public Health. (2021). 21:988. 10.1186/s12889-021-10931-5 34039306 PMC8154111

[38] Faulkner J O'Brien WJ McGrane B Wadsworth D Batten J Askew CD. Physical activity, mental health and wellbeing of adults during initial COVID-19 containment strategies: a multi-country cross-sectional analysis. J Sci Med Sport. (2021) 24:320–6. 10.1016/j.jsams.2020.11.016 33341382 PMC7711171

[39] Ali AM Kunugi H. COVID-19: a pandemic that threatens physical and mental health by promoting physical inactivity. Sport Med Head Sci. (2020) 2:221–3. 10.1016/j.smhs.2020.11.006 34189487 PMC7685939

[40] Edwardson CL Biddle SJH Clarke-Cornwell A Clemes S Davies MJ Dunstan DW. A three arm cluster randomised controlled trial to test the effectiveness and cost-effectiveness of the SMART Work & Life intervention for reducing daily sitting time in office workers: study protocol. BMC Public Health. (2018) 18:1120. 10.1186/s12889-018-6017-1 30217233 PMC6137871

[41] Dwyer MJ Pasini M De Dominicis S Righi E. Physical activity: Benefits and challenges during the COVID-19 pandemic. Scand J Med Sci Sports. (2020) 30:1291–4. 10.1111/sms.13710 32542719 PMC7323175

[42] Del Boca D Oggero N Profeta P Rossi M. Women's and men's work, housework and childcare, before and during COVID-19. Rev Econ Househ. (2020) 18:1001–17. 10.1007/s11150-020-09502-1 32922242 PMC7474798

[43] Graham M Weale V Lambert KA Kinsman N Stuckey R Oakman J. Working at home: the impacts of COVID 19 on health, family-work-life conflict, gender, and parental responsibilities. J Occup Environ Med. (2021) 63:938–43. 10.1097/JOM.0000000000002337 34325437 PMC8562911

[44] Margaria A. Fathers, Childcare and COVID-19. Fem. Leg. Stud. (2021) 29:133–44. 10.1007/s10691-021-09454-6 PMC8097245 33967411

[45] Yogman MW Eppel AM. The role of fathers in child and family health BT. engaged fatherhood for men, families and gender equality: healthcare, social policy, and work perspectives. In; Grau Grau, M., las Heras Maestro, M., Riley Bowles, H., Eds, Springer International Publishing: Cham. (2022) pp. 15–30 ISBN 978-3-030-75645-1

[46] Alon T Doepke M Olmstead-Rumsey J Tertilt M. The Impact of COVID-19 on Gender Equality. Working Paper 26947. National Bureau of Economic Research. (2020). 10.3386/w26947

[47] Mangiavacchi L Piccoli L Pieroni L. Fathers matter: Intrahousehold responsibilities and children's wellbeing during the COVID-19 lockdown in Italy. Econ Hum Biol. (2021) 42:101016. 10.1016/j.ehb.2021.101016 34044352 PMC9760207

[48] de Vries H Tummers L Bekkers V. The benefits of teleworking in the public sector: reality or rhetoric? Rev Public Pers Adm. (2019) 39:570–93. 10.1177/0734371X18760124

[49] Kamerade D Burchell B. Teleworking and participatory capital: is teleworking an isolating or a community-friendly form of work? Eur Sociol Rev. (2004) 20:345–61. 10.1093/esr/jch030

[50] Even A. The Evolution of Work: Best Practices for Avoiding Social and Organizational Isolation in Telework Employees Available online: https://ssrn.com/abstract=3543122 (accessed December 9, 2022).

[51] Mosquera P Soares ME Alvadia T. Is teleworking at odds with social sustainability and organizational learning? Learn Organ An Int J. (2022) 29:527–47. 10.1108/TLO-01-2022-0002

[52] Schaufeli WB Salanova M González-romá V Bakker AB. The measurement of engagement and burnout: a two sample confirmatory factor analytic approach. J Happiness Stud. (2002) 3:71–92. 10.1023/A:1015630930326

[53] Leo CG Sabina S Tumolo MR Bodini A Ponzini G Sabato E Mincarone P. Burnout among healthcare workers in the COVID 19 era: a review of the existing literature. Front Public Heal. (2021) 9:1661. 10.3389/fpubh.2021.750529 34778184 PMC8585922

[54] Lackritz JR. Exploring burnout among university faculty: Incidence, performance, and demographic issues. Teach Teach Educ. (2004) 20:713–29. 10.1016/j.tate.2004.07.002

[55] Padilla MA Thompson JN. Burning out faculty at doctoral research universities. Stress Heal J Int Soc Investig Stress. (2016) 32:551–8. 10.1002/smi.2661 26620490

[56] Maslach C Schaufeli WB Leiter MP. Job burnout. Annual Rev Psychol. (2001) 52:397–422. 10.1146/annurev.psych.52.1.397 11148311

[57] Özgül N Polat E. Burnout levels of academic staff: an investigation at a Public University in Turkey. Sakarya University J Sci. (2018) 22:1752–9. 10.16984/saufenbilder.39265627885969

[58] Amerio A Lugo A Stival C Fanucchi T Gorini G Pacifici R. COVID-19 lockdown impact on mental health in a large representative sample of Italian adults. J Affect Disord. (2021) 292:398–404. 10.1016/j.jad.2021.05.117 34139414 PMC8777065

[59] Skoda EM Bäuerle A Schweda A Dörrie N Musche V Hetkamp M et. al. Severely increased generalized anxiety, but not COVID-19-related fear in individuals with mental illnesses: a population based cross-sectional study in Germany. Int J Soc Psychiatry. (2021) 67:550–8. 10.1177/0020764020960773 33040668

[60] Alfonsi V Scarpelli S Gorgoni M Couyoumdjian A Rosiello F Sandroni C. Healthcare workers after 2 years of COVID-19: the consequences of the pandemic on psychological health and sleep among nurses and physicians. Int J Environ Res Public Health. (2023) 20:1410. 10.3390/ijerph20021410 36674167 PMC9859438

[61] Schmidt ME Slanger T Chang-Claude J Wahrendorf J Steindorf K. Evaluation of a short retrospective questionnaire for physical activity in women. Eur J Epidemiol. (2006) 21:575–85. 10.1007/s10654-006-9042-9 17004027

[62] Dietch JR Sethi K Slavish DC Taylor DJ. Validity of two retrospective questionnaire versions of the consensus sleep diary: the whole week and split week self-assessment of sleep surveys. Sleep Med. (2019) 63:127–36. 10.1016/j.sleep.2019.05.015 31622954

