# Corrigendum: The medium-term perceived impact of work from home on life and work domains of knowledge workers during COVID-19 pandemic: a survey at the National Research Council of Italy

**DOI:** 10.3389/fpubh.2023.1276109

**Published:** 2023-09-11

**Authors:** Antonella Bodini, Carlo Giacomo Leo, Antonella Rissotto, Pierpaolo Mincarone, Stanislao Fusco, Sergio Garbarino, Roberto Guarino, Saverio Sabina, Egeria Scoditti, Maria Rosaria Tumolo, Giuseppe Ponzini

**Affiliations:** ^1^Institute for Applied Mathematics and Information Technologies “E. Magenes”, National Research Council, Milano, Italy; ^2^Institute of Clinical Physiology, National Research Council, Lecce, Italy; ^3^Training and Welfare Unit, National Research Council, Rome, Italy; ^4^Institute for Research on Population and Social Policies, National Research Council, Brindisi, Italy; ^5^Department of Neurosciences, Rehabilitation, Ophthalmology, Genetics and Maternal/Child Sciences, University of Genoa, Genoa, Italy; ^6^Department of Biological and Environmental Sciences and Technology, University of Salento, Lecce, Italy

**Keywords:** knowledge workers, life domain, work domain, perceived impact, forced work from home, smart work

In the published article, there were a few errors in Table 1 as published. The number of women was erroneously reported (the right number is 431 rather than 425). The percentage of subjects aged 60 or older was misreported (the right percentage is 13.1% instead of 11.9%). The data relating to “Married or living together, no children” have been exchanged with those relating to “Married or living together, with children”. The number of participants with a Bachelor's degree or higher was misreported (the right number is 614 instead of 583). The corrected Table 1 and its caption appear below.

**Table 1:** Participants' general characteristics. Comparison with the source population as of December 2021, 31st, excluding managers, where feasible. Source CNR.

	**Source population n = 8**, **543**	**Sample** **n** = **748**	
	**%**	***N***	**%**
**Gender**			
Man	53.0	317	42.4
Woman	47.0	431	57.6
**Age group (years)**			
≤ 39	14.9	90	12.0
40–49	35.1	275	36.8
50–59	35.3	285	38.1
≥60	14.7	98	13.1
**Living status**			
Living alone	–	108	14.4
Married or living together, no children	–	282	37.7
Married or living together, with children	–	358	47.9
**Italian macro-region**			
North	24.0	244	32.6
Center	40.4	261	34.9
South	25.1	168	22.5
Islands	10.5	75	10.0
**Education level**			
Bachelor's degree or higher	–	614	82.1
Less than a bachelor's degree	–	134	17.9
**Professional profile**			
Administrative and technical staff	38.2	238	31.8
Researcher and technologist	61.8	510	68.2

The authors apologize for this error and state that this does not change the scientific conclusions of the article in any way. The original article has been updated.

