# Supplementary material for: The medium-term perceived impact of work from home on life and work domains of knowledge workers during COVID-19 pandemic: A survey at the National Research Council of Italy
Source: Front Public Health. 2023 Mar 10;11:1151009. doi: 10.3389/fpubh.2023.1151009 (PMC10036346; doi:10.3389/fpubh.2023.1151009)
Supplement: Supplementary file 1 [file Table_1.docx]

Supplementary Material

The medium-term perceived impact of work from home on life and work domains of knowledge workers during COVID-19 pandemic: A survey at the National Research Council of Italy

Antonella Bodini, Carlo Giacomo Leo, Antonella Rissotto^*^, Pierpaolo Mincarone, Stanislao Fusco, Sergio Garbarino, Roberto Guarino, Saverio Sabina, Egeria Scoditti, Maria Rosaria Tumolo, Giuseppe Ponzini

*** Correspondence:** Antonella Rissotto, antonella.rissotto@cnr.it

# Supplementary Figures and Tables

## Supplementary Figure 1


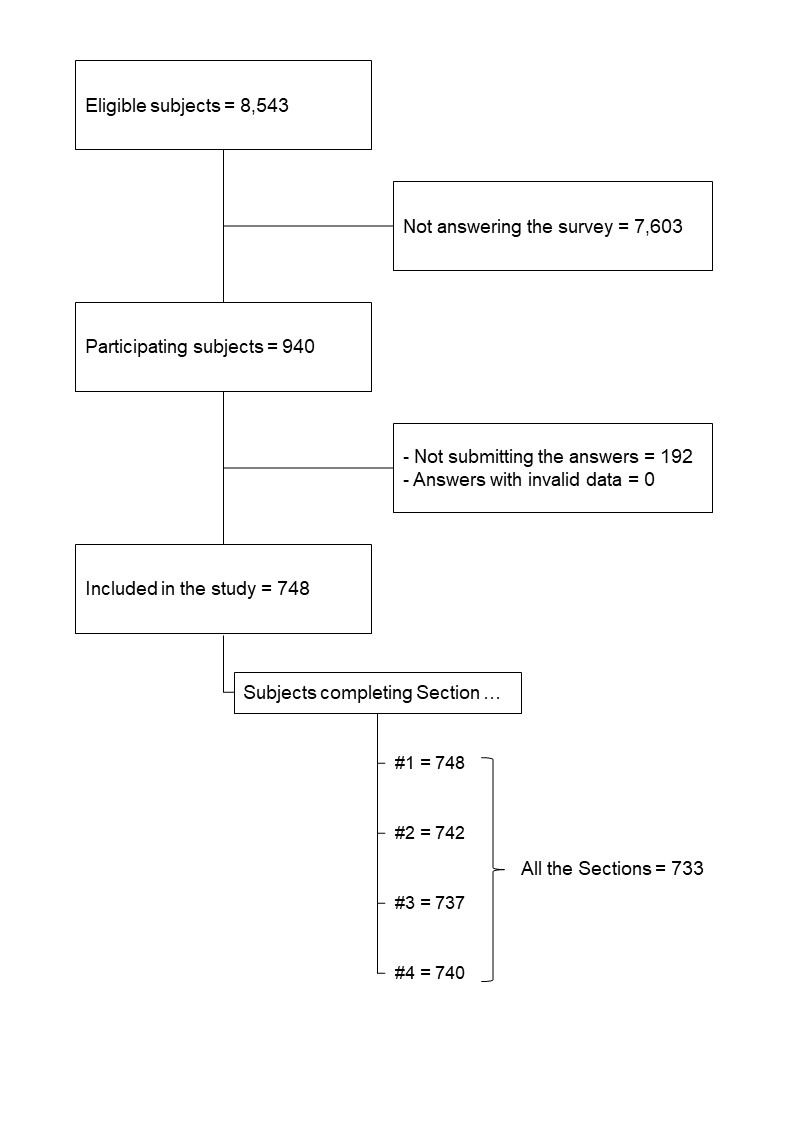


**Supplementary Figure 1.** Flow chart of study participants.

## Supplementary Figure 2

| 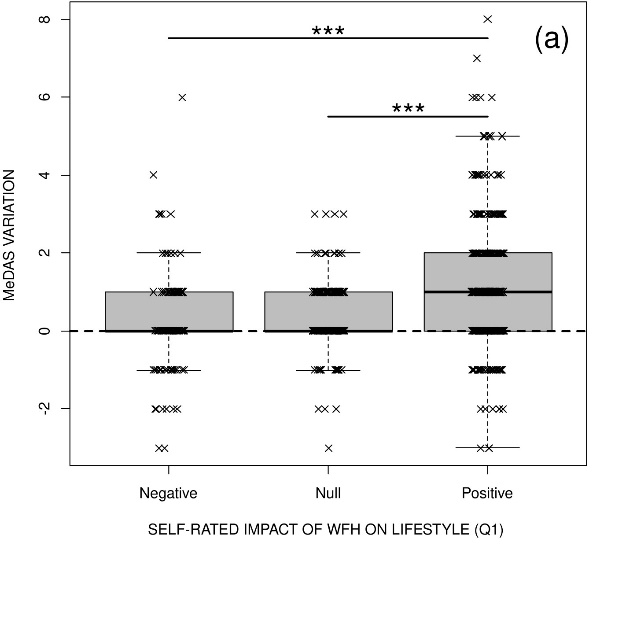 | 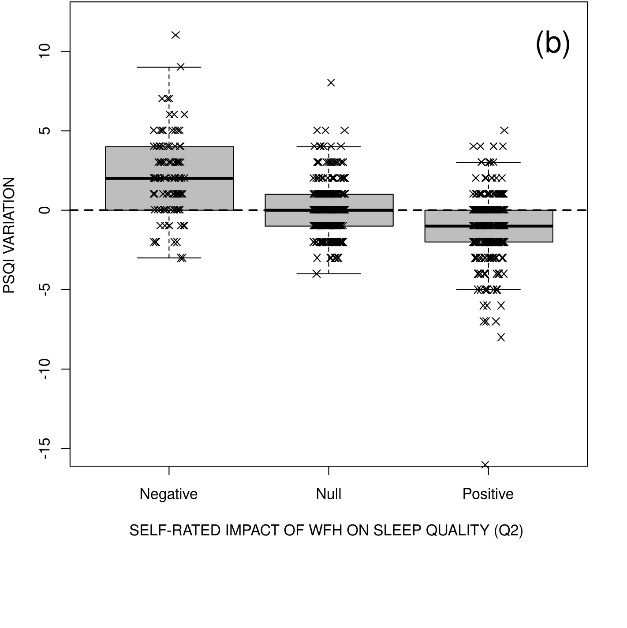 |
| --- | --- |
| 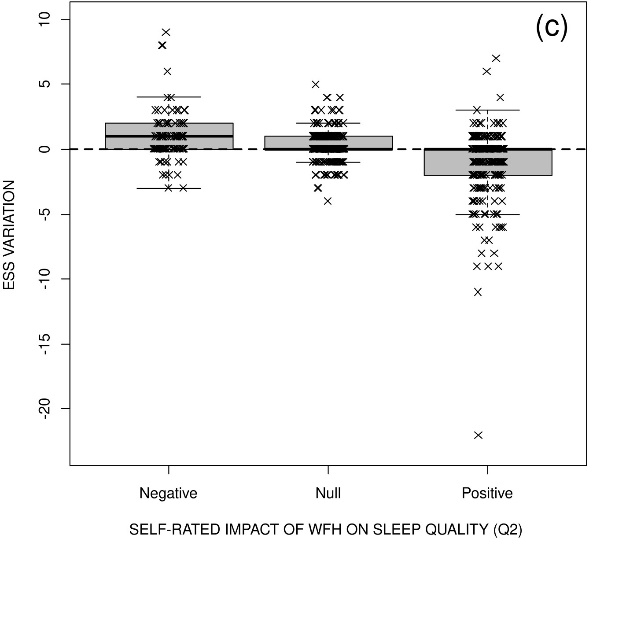 | 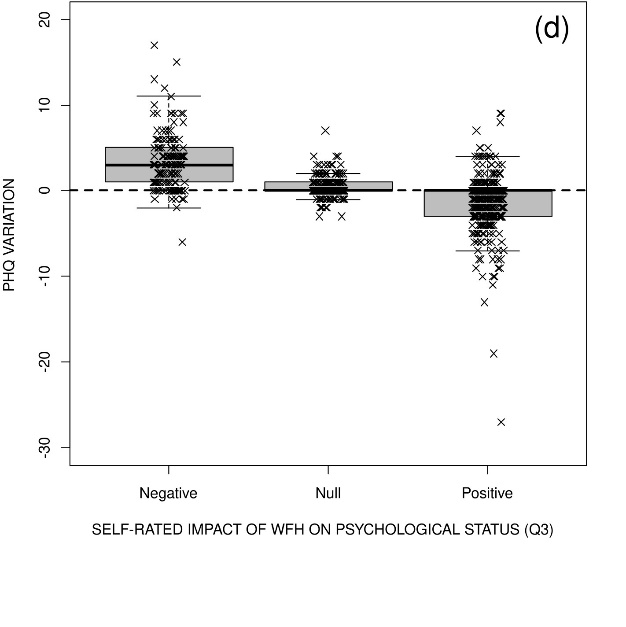 |

**Supplementary Figure 2.** Variation (during WFH-before WFH) of MeDAS (a), PSQI (b), ESS (c) and PHQ (d) total scores compared to (Q1)-(Q3) self-rated changes. All the *p*-values of the Kruskal-Wallis tests are $\ll0.001$ and all the pairwise comparisons were statistically significant (Bonferroni-adjusted *p*-values $< 0.001$) except in the case of MeDAS vs Q1 analysis, as shown in Figure (a) by usual notations (segment and asterisks).

## Supplementary Tables

Supplementary Table 1. Contingency tables for the cross-check of the reliability of the self-assessed rates in (Q1)-(Q3) of the life domain and *p*-values of chi-square test.

| \| $p<0.001$ \| **MeDAS variation^2^** \| \| \| \| --- \| --- \| --- \| --- \| \| **Q1^1^** \| Worsening \| No change \| Improvement \| \| Negative \| 26 \| 55 \| 36 \| \| Null \| 18 \| 111 \| 69 \| \| Positive \| 41 \| 172 \| 214 \| | \| $p=0$ \| **PSQI variation^2^** \| \| \| \| --- \| --- \| --- \| --- \| \| **Q2^1^** \| Worsening \| No change \| Improvement \| \| Negative \| 73 \| 14 \| 14 \| \| Null \| 98 \| 142 \| 117 \| \| Positive \| 47 \| 66 \| 166 \| |
| --- | --- | --- | --- | --- | --- | --- | --- | --- | --- | --- | --- | --- | --- | --- | --- | --- | --- | --- | --- | --- | --- | --- | --- | --- | --- | --- | --- | --- | --- | --- | --- | --- | --- | --- | --- | --- | --- | --- | --- | --- | --- |
| \| $p=0$ \| **ESS variation^2^** \| \| \| \| --- \| --- \| --- \| --- \| \| **Q2^1^** \| Worsening \| No change \| Improvement \| \| Negative \| 53 \| 36 \| 12 \| \| Null \| 94 \| 216 \| 47 \| \| Positive \| 54 \| 101 \| 124 \| | \| $p=0$ \| **PHQ variation^2^** \| \| \| \| --- \| --- \| --- \| --- \| \| **Q3^1^** \| Worsening \| No change \| Improvement \| \| Negative \| 119 \| 23 \| 7 \| \| Null \| 69 \| 130 \| 28 \| \| Positive \| 67 \| 120 \| 177 \| |
| ^1^ Very negative (very positive) responses to (Q1)-(Q3) were added to the negative (positive) ones to obtain three categories of negative, null and positive perceived impact*.*  ^2^ Worsening: decrease in MeDAS score, increase in PSQI, ESS, and PHQ scores during WFH compared to pre-WFH. Improvement: increase in MeDAS score, decrease in PSQI score, ESS and PHQ scores during WFH compared to pre-WFH. | |

**Supplementary Table 2.** Multivariable multinomial logistic regression analysis results for the items of the life domain. Statistically significant ORs and their 95% CIs are reported in bold. For each item in the life domain, the two columns report the ORs and their 95% CIs of the two comparisons of the outcomes of negative perception and of positive perception with the reference outcome of none perceived impact, respectively.

|  | **Q1**  **lifestyle & health** | | **Q2**  **sleep quality** | | **Q3**  **psychological status** | | **Q4/5**  **family & friends** | |
| --- | --- | --- | --- | --- | --- | --- | --- | --- |
|  | **Negative vs**  **None**  OR; 95% CI | **Positive vs**  **None**  OR; 95% CI | **Negative vs**  **None**  OR; 95% CI | **Positive vs**  **None**  OR; 95% CI | **Negative vs**  **None**  OR; 95% CI | **Positive vs**  **None**  OR; 95% CI | **Negative vs**  **None**  OR; 95% CI | **Positive vs**  **None**  OR; 95% CI |
| **Variable** |  |  |  |  |  |  |  |  |
| **Age class** | | | | | | | | |
| < 39 | 1.00 | 1.00 | 1.00 | 1.00 |  |  | 1.00 | 1.00 |
| 40-49 | 0.45;  0.17-1.18 | **0.38;**  **0.18-0.82** | 1.14;  0.49-2.63 | **0.53;**  **0.30-0.93** |  |  | 1.41;  0.66-3.00 | **0.52;**  **0.30-0.92** |
| 50-59 | **0.33;**  **0.12-0.88** | **0.31;**  **0.15-0.68** | 1.14;  0.49-2.64 | 0.60;  0.34-1.06 |  |  | 1.88;  0.88-4.01 | 0.60; 0.34-1.05 |
| ≥ 60 | **0.14;**  **0.04-0.46** | **0.13;**  **0.06-0.32** | 0.44;  0.14-1.36 | **0.24;**  **0.12-0.49** |  |  | 1.31;  0.53-3.24 | **0.25;**  **0.12-0.53** |
| **Living alone** | | | | | | | | |
| No |  |  | 1.00 | 1.00 | 1.00 | 1.00 |  |  |
| Yes |  |  | **2.41; 1.22-4.74** | 1.15; 0.68-1.96 | **3.97; 1.97-8.02** | 1.71; 0.95-3.08 |  |  |
| **Size of the city of residence (in thousands of inhabitants)** | | | | | | | | |
| <15 | 1.00 | 1.00 |  |  |  |  |  |  |
| 15-50 | 0.54; 0.23-1.30 | 0.70; 0.40-1.21 |  |  |  |  |  |  |
| 50-100 | 1.37; 0.55-3.41 | 1.06; 0.56-2.00 |  |  |  |  |  |  |
| >100 | 1.73; 0.86-3.47 | 0.94; 0.58-1.52 |  |  |  |  |  |  |
| **Professional profile in the CNR** | | | | | | | | |
| Researcher |  |  | 1.00 | 1.00 |  |  | 1.00 | 1.00 |
| Technologist |  |  | 1.06;  0.46-2.41 | 1.50;  0.85-2.63 |  |  | 0.76;  0.37-1.57 | **2.02;**  **1.13-3.60** |
| Administrative  staff |  |  | **2.32;**  **1.01-5.32** | **3.12;**  **1.71-5.70** |  |  | 0.91;  0.42-1.95 | **2.13;**  **1.19-3.79** |
| Technician |  |  | 1.26; 0.70-2.26 | 1.21; 0.79-1.86 |  |  | 0.92; 0.55-1.55 | 1.51; 0.96-2.36 |
| **Graduation** | | | | | | | | |
| No |  |  |  |  | 1.00 | 1.00 |  |  |
| Yes |  |  |  |  | 1.31;  0.66-2.60 | **0.55;**  **0.34-0.87** |  |  |
| **Availability of a fixed workstation (room, table, pc, printer etc.) at home** | | | | | | | | |
| No |  |  |  |  | 1.00 | 1.00 | 1.00 | 1.00 |
| Yes |  |  |  |  | 0.85; 0.51-1.44 | 1.39; 0.91-2.11 | 0.53; 0.33-0.83 | 1.04; 0.67-1.61 |
| **Frequency of sharing the work room at home** | | | | | | | | |
| Never | 1.00 | 1.00 | 1.00 | 1.00 | 1.00 | 1.00 | 1.00 | 1.00 |
| Occasionally | 0.80; 0.43-1.50 | 0.80; 0.52-1.23 | 1.19; 0.64-2.23 | 0.77; 0.51-1.17 | 1.60; 0.88-2.91 | 1.15; 0.75-1.76 | 1.10; 0.66-1.82 | 0.81; 0.53-1.22 |
| Often or  always | 1.65;  0.89-3.05 | 0.67;  0.41-1.09 | **2.53;**  **1.35-4.75** | 0.66;  0.41-1.05 | **3.09;**  **1.67-5.72** | 1.08;  0.66-1.75 | **1.96;**  **1.17-3.27** | **0.55;**  **0.33-0.91** |
| **Need to provide assistance to cohabitants** | | | | | | | | |
| No |  |  |  |  | 1.00 | 1.00 |  |  |
| Yes |  |  |  |  | 1.76; 0.88-3.53 | 1.80; 1.03-3.15 |  |  |
| **Time taken to get from home to work (in minutes)** | | | | | | | | |
| ≤ 15 | 1.00 | 1.00 | 1.00 | 1.00 | 1.00 | 1.00 | 1.00 | 1.00 |
| 15-30 | 1.15;  0.58-2.26 | **2.26;**  **1.32-3.84** | 0.55;  0.29-1.05 | **2.19;**  **1.29-3.72** | 0.78;  0.42-1.44 | 1.33;  0.80-2.23 | 0.58;  0.33-1.01 | 1.29;  0.75-2.22 |
| 30-60 | 1.66;  0.84-3.26 | **2.82; 1.63-4.89** | 0.63;  0.34-1.16 | **1.86;**  **1.10-3.16** | 0.87;  0.47-1.61 | 1.39;  0.83-2.33 | 0.69;  0.40-1.18 | 1.24;  0.72-2.14 |
| > 60 | 0.62;  0.25-1.54 | **2.44;**  **1.32-4.48** | 0.54;  0.24-1.23 | **3.33;**  **1.85-5.99** | 0.68;  0.31-1.50 | **2.57;**  **1.40-4.69** | **0.38;**  **0.18-0.80** | **2.37;**  **1.32-4.27** |
| **Number of days of work in presence during the WFH period** | | | | | | | | |
| < 20 | 1.00 | 1.00 |  |  |  |  |  |  |
| 21-60 | 0.62; 0.31-1.24 | 0.83; 0.52-1.34 |  |  |  |  |  |  |
| 61-120 | 1.24; 0.63-2.42 | 0.78; 0.46-1.30 |  |  |  |  |  |  |
| > 120 | 0.56; 0.27-1.15 | 0.45; 0.27-0.76 |  |  |  |  |  |  |
| **Hobbies/pastimes** | | | | | | | | |
| Neither before nor during WFH | 1.00 | 1.00 | 1.00 | 1.00 | 1.00 | 1.00 | 1.00 | 1.00 |
| Before no and during WFH yes | 0.52;  0.12-2.33 | **4.22;**  **1.64-10.8** | 1.56;  0.48-5.10 | **3.59;**  **1.68-7.68** | 0.43;  0.10-1.74 | **4.31;**  **1.91-9.73** | 1.14;  0.42-3.12 | **3.01;**  **1.40-6.45** |
| Before yes and during WFH no | 1.86;  0.67-5.10 | 1.18;  0.49-2.87 | 2.27;  0.87-5.97 | 1.38;  0.60-3.16 | **5.00;**  **1.79-14.0** | **3.94;**  **1.47-10.6** | 2.22;  0.95-5.18 | 1.74;  0.74-4.10 |
| Both before and during WFH | 1.00;  0.48-2.08 | 1.44;  0.80-2.58 | 1.54;  0.73-3.26 | 1.50;  0.88-2.55 | 1.36; 0.70-2.66 | **2.05;**  **1.16-3.61** | 1.36;  0.72-2.57 | 1.49;  0.84-2.64 |
| **Practicing vigorous physical activity** | | | | | | | | |
| Discontinuation during WFH due to restrictions | 1.00 | 1.00 |  |  |  |  |  |  |
| No habit | **0.37; 0.18-0.75** | 0.78; 0.43-1.43 |  |  |  |  |  |  |
| Regularly during WFH | **0.32; 0.14-0.76** | 0.83; 0.43-1.59 |  |  |  |  |  |  |
| **Practicing moderate physical activity** | | | | | | | | |
| Discontinuation during WFH due to restrictions | 1.00 | 1.00 |  |  | 1.00 | 1.00 |  |  |
| No habit | 1.34; 0.59-3.04 | 1.16; 0.58-2.29 |  |  | 0.94; 0.48-1.85 | 1.53; 0.84-2.79 |  |  |
| Regularly during WFH | 0.89;  0.42-1.88 | 1.83;  0.98-3.42 |  |  | 0.69;  0.38-1.28 | **1.83;**  **1.05-3.19** |  |  |
| **Sedentary Lifestyle** | | | | | | | | |
| Decreased | 1.00 | 1.00 | 1.00 | 1.00 | 1.00 | 1.00 | 1.00 | 1.00 |
| Unchanged | **0.25;**  **0.08-0.73** | **0.25;**  **0.12-0.52** | 0.40;  0.15-1.11 | **0.38;**  **0.22-0.66** | **0.22;**  **0.09-0.56** | **0.27;**  **0.14-0.52** | 0.72;  0.32-1.59 | **0.53;**  **0.31-0.89** |
| Increased | 0.72;  0.26-2.02 | **0.22;**  **0.10-0.47** | 1.64;  0.66-4.07 | **0.26;**  **0.15-0.46** | 0.78; 0.33-1.84 | **0.24;**  **0.12-0.47** | 1.69;  0.79-3.59 | **0.30;**  **0.17-0.52** |
| Much increased | 1.27;  0.40-4.03 | **0.24;**  **0.10-0.59** | 2.04;  0.74-5.64 | **0.22;**  **0.10-0.47** | 1.42;  0.52-3.86 | **0.26;**  **0.11-0.61** | **2.58;**  **1.08-6.14** | **0.32;**  **0.15-0.68** |
|  | **Negative vs**  **None**  OR; 95% CI | **Positive vs**  **None**  OR; 95% CI | **Negative vs**  **None**  OR; 95% CI | **Positive vs**  **None**  OR; 95% CI | **Negative vs**  **None**  OR; 95% CI | **Positive vs**  **None**  OR; 95% CI | **Negative vs**  **None**  OR; 95% CI | **Positive vs**  **None**  OR; 95% CI |

Supplementary Table 3. Estimated proportional odds model expressed as $\boldsymbol{P}\left( \boldsymbol{Y\leq k} \right)\boldsymbol{=}\boldsymbol{\alpha}_{\boldsymbol{k}}\boldsymbol{-}\left( \boldsymbol{\beta}_{\boldsymbol{1}}\boldsymbol{x}_{\boldsymbol{1}}\boldsymbol{+\cdots}\boldsymbol{\beta}_{\boldsymbol{p}}\boldsymbol{x}_{\boldsymbol{p}} \right)$. According to the R’s parameterization of the model, a positive coefficient ($\boldsymbol{\beta}$) corresponds to WEM being more likely to fall at the high end of the scale as the levels of the factor “increases”, (1). In other words, the reported ORs refer to the outcomes high WEM versus a lower WEM.

| **Variable** | **Coef (**$\boldsymbol{\beta}$**)** | **standard**  **error** | **p-value** | **OR (**$\mathbf{e}^{\boldsymbol{\beta}}$**)^1^** | **OR**  **95% CI** |
| --- | --- | --- | --- | --- | --- |
| **Availability of a work room** |  |  |  |  |  |
| No | -- | -- | -- | 1.00 | -- |
| Yes | 0.448 | 0.169 | 0.008 | 1.56* | 1.12-2.18 |
| **Sharing the work room** |  |  |  |  |  |
| Never | -- | -- | -- | 1.00 | -- |
| Occasionally | -0.039 | 0.168 | 0.80 | 0.96 | 0.69-1.34 |
| Often or always | -0.573 | 0.186 | 0.002 | 0.56* | 0.39-0.81 |
| **Time to go from home to work** |  |  |  |  |  |
| <15 min | -- | -- | -- | 1.00 | -- |
| 15-30 min | 0.626 | 0.209 | 0.003 | 1.87* | 1.24-2.82 |
| 30-60 min | 0.819 | 0.206 | <0.001 | 2.27* | 1.52-3.40 |
| >60 min | 1.377 | 0.240 | <0.001 | 3.96* | 2.48-6.37 |
| **Number of days in the office** |  |  |  |  |  |
| <20 | -- | -- | -- | 1.00 | -- |
| 21-60 | 0.019 | 0.182 | 0.90 | 1.02 | 0.71-1.46 |
| 61-120 | -0.168 | 0.198 | 0.40 | 0.84 | 0.57-1.24 |
| >120 | -0.484 | 0.212 | 0.023 | 0.62* | 0.40-0.93 |
| **Professional level** |  |  |  |  |  |
| Researcher | -- | -- | -- | 1.00 | -- |
| Technologist |  |  |  | 1.48 | 0.92-2.37 |
| Administrative staff |  |  |  | 2.33* | 1.42-3.88 |
| Technician |  |  |  | 1.01 | 0.71-1.45 |
| **Assistance to non-cohabitants** |  |  |  |  |  |
| No | -- | -- | -- | 1.00 | -- |
| Yes | 0.311 | 0.156 | 0.046 | 1.36* | 1.01-1.85 |
| **Macro-region of residence** |  |  |  |  |  |
| North | -- | -- | -- | 1.00 | -- |
| Center | 0.130 | 0.175 | 0.50 | 1.14 | 0.81-1.60 |
| South | 0.099 | 0.195 | 0.60 | 1.10 | 0.75-1.62 |
| Islands | 0.712 | 0.260 | 0.006 | 2.04* | 1.23-3.41 |
| **Sedentary lifestyle** |  |  |  |  |  |
| Decreased | -- | -- | -- | 1.00 | -- |
| Unchanged | 0.030 | 0.233 | 0.90 | 1.03 | 0.65-1.62 |
| Increased | -0.919 | 0.234 | <0.001 | 0.40* | 0.25-0.63 |
| Much increased | -1.366 | 0.303 | <0.001 | 0.26* | 0.14-0.46 |
| **Intercepts** |  |  |  |  |  |
| Negative vs Moderately Positive or Positive | -0.354 | 0.362 | 0.30 | -- | -- |
| Negative or Moderately Positive vs Positive | 1.372 | 0.366 | <0.001 | -- | -- |
| ^1^ The odds ratios under 1 are inverted to interpret the odds ratios rationally.  * Statistically significant OR. | | | | | |

**References:**

1. Venables, W.N.; Ripley, B.D. *Modern Applied Statistics with S. Fourth Edition*; Springer, 2002; ISBN 0-387-95457-0, 2002
